# Navigating Through the Caregiving Trajectory: A Qualitative Interview Study on the Challenges Encountered by Primary Caregivers When Caring for Adolescents Living with HIV

**DOI:** 10.3390/nursrep16090308

**Published:** 2026-08-30

**Authors:** Jeaneth Linki Matsimbi, Maditobane Robert Lekganyane

**Affiliations:** Department of Social Work, University of South Africa, Pretoria 0028, South Africa; matsijl@unisa.ac.za

**Keywords:** HIV, adolescent living with HIV, primary caregiver, experiences, challenges

## Abstract

**Background/Objectives:** Children and adolescents living with HIV (ALHIV) are among the most vulnerable groups affected by HIV and AIDS. Their vulnerability exposes them to several challenges, such as HIV-related stigma and/or interrupted academic activities due to health-related complications. Since they are still minors who cannot independently make decisions regarding their own lives, most of the challenges extend to their caregivers, who sometimes provide care and support under difficult psychological, social, and economic conditions. This study aimed to explore the lived experiences and challenges of primary caregivers caring for ALHIV in Gauteng Province of South Africa. **Methods:** A qualitative exploratory design with phenomenological orientation was adopted. Eight primary caregivers of ALHIV were purposively sampled and interviewed using individual face-to-face semi-structured interviews. Data was analysed thematically according to the eight steps of Tesch’s qualitative analysis framework. Since it involved human participants, this study was subjected to ethical clearance by the Research Ethics Committee of the University of South Africa, which imposed conditions for upholding ethical principles such as informed consent and confidentiality. **Results:** Primary caregivers encounter challenges such as regular monitoring of ALHIV who are always sick, treatment adherence monitoring, behavioural management, financial strain, stigma and discrimination, lack of proper nutrition and managing adolescents’ academic difficulties. These challenges require support from multidisciplinary team members which include nurses because they play a crucial role in supporting treatment adherence among ALHIV and in strengthening the capacity of primary caregivers to provide effective treatment support. **Conclusions:** It is essential to adopt an integrated approach to psychosocial, as well as financial support interventions targeting primary caregivers.

## 1. Introduction

HIV remains a major global public health concern, with statistical estimates reporting that 40.8 million people were living with it in 2024. Moreover, some countries are still reporting increases in new infections, with an estimated 1.3 million people having been infected with HIV in 2024 [1]. Being among the most vulnerable groups, adolescents living with HIV (ALHIV) are estimated to be around 1.57 million globally, of whom only 64% were receiving life-saving antiretroviral therapy (ART) in 2024. Should the current trends continue unabated, it is estimated that 184,000 adolescents will be infected with HIV every year [2]. As part of the global community, South Africa is not immune to the phenomenon of HIV and its related challenges. During the year 2024, South Africa was estimated to have approximately 7.8 million people living with HIV (PLHIV), with about 170,000 having been newly infected [3]. Regarding adolescents in South Africa, it was projected in 2022 that those living with HIV were around 360,000 [4].

Several studies [5,6,7,8,9] indicate that ALHIV are facing various physical and psychosocial challenges, including frequent illnesses, difficulties in disclosing their HIV diagnoses, failure to regularly take treatment, stigmatisation, and engaging in risky sexual behaviours, as well as cognitive and learning difficulties. Accordingly, the South African National AIDS Council (SANAC) revealed that adolescents continue to lag in AIDS responses compared to adults, a challenge that is putting too many young lives at risk of sickness and death [10].

Like any other person, ALHIV do not live their lives in isolation. They are part of families, neighbourhoods, and general society. Issues such as poverty, unemployment, crime, and environmental disasters bear an impact on them and their primary caregivers. Among the compounding challenges faced by ALHIV is their vulnerability as minors due to their dependence on adults for guidance and support on certain health-related decisions. This implies that the challenges experienced by ALHIV should not be considered in isolation because they also have a bearing on their primary caregivers. Moreover, the disease burden among adolescents has negatively affected the financial, physical, and psychological well-being of their primary caregivers, which in turn affects the quality of life of these ALHIV [11]. Primary caregivers in the context of this study refer to biological parents, foster parents, adoptive parents, grandparents, legal guardians or any other person who is responsible for the needs of the adolescent including the provision of care, support and shelter.

This study sought to amplify the voices of caregivers of ALHIV by exploring their lived experiences and challenges when caring for ALHIV in Gauteng Province, South Africa. Although previous studies [9,12,13,14,15] have explored the experiences of caregivers of ALHIV, limited research has examined how their challenges across individual, family, community, and healthcare system levels shape caregiving experiences within the South African context. This knowledge gap limits the development of context-specific support interventions; hence, our study focused on this subject matter.

## 2. The Socioeconomic and Psychosocial Impact of HIV on ALHIV and Their Primary Caregivers

HIV affects the individual person, their families, their communities, and many other aspects of human life in several ways. It does not only have devastating effects on the individual’s health, but also presents substantial socioeconomic challenges to individuals, families, communities, and societies. To adolescents and their caregivers, it poses a tremendous threat. Sometimes these caregivers care for adolescents with special needs, and often without the necessary resources to address them. The socioeconomic status of ALHIV and their primary caregivers may equally be compromised because HIV creates additional needs that can result in poverty due to loss of work and depleted family income, the costs of caring for sick family members and orphaned children, weakened family support systems, exhausted social resources, decreased participation in formal education by adolescents, and a reduction in development and national economic growth [14,15,16].

HIV also presents psychosocial challenges to ALHIV and their families. ALHIV experience numerous psychosocial and emotional challenges, such as accepting and coping with an HIV diagnosis, taking HIV treatment, disclosing their HIV-positive diagnosis, and loss of parents or insufficient care from primary caregivers, as well as seeking socio-emotional support [17,18]. Due to the discriminatory attitudes regarding HIV, ALHIV often lack the necessary social support which has the potential to result in poor self-esteem, stigma and discrimination. This may predispose them to psychological problems, such as depression and anxiety. Moreover, living with HIV brings about long-term stress due to the life-threatening nature of the disease, the treatment’s side-effects, and the internal and external stigma associated with it [19,20]. Furthermore, it has been established that ALHIV suffer from mental health-related issues that, in turn, aggravate their general state of health [21,22,23]. Primary caregivers also encounter challenges of having to care for children and adolescents who have lost their biological parents due to AIDS-related conditions. Some of these children are exposed to depression, anxiety, loneliness, anger, fear, and stigmatisation by virtue of being associated with their deceased parents, a situation which weighs heavily on their caregivers [22,24,25].

## 3. Nurses as a Support System for Adolescents Living with HIV

Despite the challenges encountered by both primary caregivers and the adolescents in their care, they draw strength from various support structures including nurses who play a prominent role. As frontline healthcare professionals, nurses are generally responsible for supporting children and adolescents through adherence counselling, health education, monitoring treatment outcomes, identifying barriers to medication adherence, and facilitating linkages to psychosocial and community-based support services [26,27]. They also assist adolescents and caregivers in navigating challenges such as treatment fatigue, stigma, disclosure challenges, forgetfulness, and limited social support, all of which may negatively affect adherence to ART [28,29]. Nurses are specifically responsible for assessing the adolescents’ ages and maturation, development and developmental milestones, menarche (first menstrual periods), genetic inheritance, physiological function and herd immunity and determine the necessary interventions. Regarding development and developmental milestones, nurses would assess the developmental delays and the extent to which specific milestones are achieved [30]. Nurses acknowledge that, in the process of their growth and development, these adolescents encounter various difficulties including those that involve an environment which nurtures development. In such instances, nurses would liaise with the caregivers to ensure that adolescents have an environment which stimulates their growth and development.

Since part of the adolescents’ development involves relationship issues with their caregivers, nurses also play a supportive role of assuring caregivers that it is normal for adolescents to test family values, and such does not necessarily mean they are rebellious. As part of their support to these adolescents, nurses also consider the menarche as well as other physical and emotional changes by assessing the extent of sexual changes and knowledge of menstruation as well as preparation thereof [30]. Through regular interaction with adolescents and their families, nurses are positioned to promote treatment literacy, encourage retention in care, and foster collaborative relationships with primary caregivers that support sustained adherence [31]. Understanding the experiences and challenges faced by both the primary caregivers and adolescents is essential for informing nursing interventions and developing family-centred approaches that improve treatment outcomes and quality of life for ALHIV.

## 4. An Ecological Theoretical Perspective on the Primary Caregivers of ALHIV

A theoretical framework is a guide supported by a specific theory or theories that the researcher uses to plan their research study and should, therefore, be clarified at the inception of the study [32]. This study was guided by ecological systems theory, which provides a framework for understanding how individuals interact with and are influenced by their social and physical environments [33,34,35]. The theory conceptualises human development as occurring through dynamic interactions across five interconnected systems: the microsystem, mesosystem, exosystem, macrosystem, and chronosystem [33,34,36,37].

Within the context of this study, the microsystem represents the immediate environment in which ALHIV receive care and support, primarily from their caregivers [33,34,37]. The mesosystem reflects the interactions between these immediate settings, such as collaboration among caregivers, schools, healthcare providers, and social workers to promote adolescents’ well-being, treatment adherence, and educational outcomes. The exosystem comprises external structures that indirectly influence ALHIV, including caregivers’ workplaces and organisational policies that may affect their ability to provide consistent care to ALHIV as well as access to healthcare services [33,34,36,37].

The use of ecological systems theory in this study is based on the theory’s focus on how the experiences and challenges of primary caregivers are shaped by the dynamic interactions across multiple environmental systems including family, schools, healthcare services, workplaces, communities and wider social and policy contexts [38]. This holistic perspective informed the exploration of caregivers’ experiences and challenges and therefore guided the interpretation of the study’s findings.

## 5. Materials and Methods

A qualitative exploratory design with a phenomenological orientation was adopted to explore the lived experiences and challenges of primary caregivers caring for ALHIV in Gauteng Province, South Africa. This approach enabled the collection of rich, contextual data in the natural settings of the participants, such as their homes, NPOs, or clinics. Qualitative research was chosen for its ability to enable the development of knowledge regarding participants’ lived experiences, which is important to understanding and addressing social issues or problems such as HIV among adolescents [39,40]. Problems or issues need to be explored by focusing on participants’ perspectives, the meanings they attach to the phenomenon under investigation, and their multiple subjective views that are all compatible with qualitative research [41]. In line with qualitative research, participants’ perspectives, ideas, and subjective experiences were explored through semi-structured interviews.

### 5.1. Study Setting

The study was conducted in four municipalities of South Africa’s Gauteng province, namely Johannesburg, Ekurhuleni, Sedibeng, and Tshwane. The choice of these study sites was influenced by time and financial constraints, as well as the prevalence of HIV. As reported by some researchers [42], Gauteng province is the second most affected province, with an estimated total of 66,754 adolescents reported to be living with HIV.

### 5.2. Population and Sampling

Participants were recruited from the Department of Social Development (DSD)-funded non-profit organisations (NPOs) that were providing services to orphans, vulnerable children, adolescents, and youth (OVCA&Y). Access to these organisations was facilitated by an organisation called HIV South Africa (HIVSA), and permission to conduct the study was obtained from NPO managers who served as gatekeepers. Community caregivers and social workers assisted in identifying eligible participants and facilitating contact with those who had consented to be approached. The researcher subsequently met with potential participants to introduce the study, obtain written informed consent, and prepare for the interviews. Purposive sampling was used to recruit primary caregivers of ALHIV. Participants were eligible for participation if they were (1) a primary caregiver of an ALHIV, (2) aware of the adolescent’s HIV-positive status, (3) willing to participate, and (4) receiving services from DSD-funded NPOs within the city municipalities of Johannesburg, Sedibeng, Tshwane, and Ekurhuleni. Although recruitment was challenging because of the sensitive nature of the topic, eight primary caregivers met the inclusion criteria and participated in the study. Data was analysed immidiately after each interview. New text and ideas were constantly compared with older analysed data to see if fresh concepts appeared. This process continued until the eighth interview when no new information emerged, which then signalled that data saturation was achieved [43,44].

### 5.3. Data Collection

Data collection in qualitative research is a process of collecting information about the participants’ realities to better understand their experiences and eventually answer specific research questions [45]. Observations, interviews, and focus group discussions are commonly used methods of data collection in qualitative studies [41]. For the purpose of this study, the data collection method was individual semi-structured face-to-face interviews that were supported by an interview guide. The questions in the guide were open-ended, phrased in a flexible and neutral fashion, and supplemented by follow-up probing questions based on the participants’ responses. The interview guide was informed by the research questions, goals, and objectives of the study. The guide comprised two parts, namely demographical questions (which required participants to share their age, gender, education, and employment status) and key questions about the subject of research (including what are the experiences and challenges of primary caregivers in relation to caring for ALHIV). Data collection started in July 2023 and ended in July 2024, with each interview lasting for approximately 40 to 60 min. The interviews were conducted in the primary residence of primary caregivers, as per their preferences. For some participants, due to non-disclosure and a lack of privacy in their homes, the interviews were conducted at the NPOs or the healthcare facilities where they accessed services. Interviews were conducted in Setswana, Isizulu, Xitsonga and English. The interviews that were conducetd in Xitsongs and Setswana languages were later translated into English by the researchers who were both conversant in the two languages. All interviews were audio-recorded with permission of the participants and transcribed word-for-word prior to analysis. The interviews were conducted by the researcher (author 1), and the supervisor (author 2) oversaw the process. Drawing on over 20 years of professional experience as a social worker in the HIV field and a strong background in qualitative research at both master’s and doctoral levels, researchers reflected continuously on how their knowledge and experiences influenced data collection and interpretation. To enhance reflexivity, researchers maintained reflective notes documenting their thoughts, feelings, and experiences throughout the research process, thereby strengthening the trustworthiness and credibility of the study’s findings [46,47].

### 5.4. Data Analysis

Data were analysed by employing Tesch’s framework of thematic analysis, which included coding, categorisation, and iterative theme development to ensure alignment with the research objectives, therefore making it easier to describe the phenomenon under investigation. The researcher (author1) was involved in the coding process and the supervisor (author 2) played an oversight role. No interviews were repeated in this study because there were no gaps that necessitated interviews to be repeated. To enhance rigour, the study adopted Guba and Lincoln’s strategies of credibility, confirmability, dependability, and transferability [46,48,49]. To implement these principles, the following measures were adopted:

- Credibility was enhanced through iterative questioning and probing during interviews. Honest engagement with participants, peer scrutiny, researcher expertise, and triangulation of data sources (i.e., interviews with primary caregivers from different organisations, of different age groups and gender) [50,51]. Literature was used from different disciplinary backgrounds such as social work, sociology, psychology and health sciences, and two researchers were involved in this study from the planning to reporting stages, with one playing an oversight role. Memberchecking was also upheld by taking the interview transcripts to participants to verify if they indeed reflected their experiences [50,51,52].
- Transferability was promoted through thick description of the research context, participants, and data collection process, enabling readers to determine the applicability of the findings to similar settings [50,51,52].
- Dependability was achieved by maintaining a comprehensive audit trail documenting data collection, transcription, coding, and analysis procedures, and consensus discussions between the researcher and supervisor [50,51,52].
- Confirmability was strengthened through accurate record keeping, audio-recording and verbatim transcription of interviews, maintenance of an audit trail, peer debriefing, triangulation, and the use of direct participant quotations to support the study findings [50,51,52].

### 5.5. Ethical Considerations

This study involved human participants and therefore required ethical clearance, which was obtained from the University of South Africa’s College of Human Science Research Ethics Committee (CREC) (31368018_CREC_CHS_2024). The involvement of human participants in this study required compliance with the ethical principles of obtaining informed consent, upholding confidentiality and anonymity, avoiding harm, and promoting beneficence, as well as securing data [43,53]. To uphold confidentiality and anonymity, pseudonyms were used, and the principle of no harm entailed maximising benefits and minimising risks by not pressing for any sensitive questions that would result in emotional strain on the participants while contributing minimally to the overall study aim [54,55,56]. On securing data, all of the participants’ data were safely and privately kept in a lockable cabinet in Author One’s home office, where she was the only person with access. All the electronic data were password-protected in both authors personal laptops [57].

## 6. Results

The results of this study are in two parts. The first part addresses the demographic profiles of the participants, and the second part focuses on the substantive part of the research findings.

### 6.1. Demographics of the Participants

Part of the research findings were based on the demographic data which was collected through six questions which required participants to share their demographic information such as age, gender, ethnicity, their education level, employment status, and their relationship with ALHIV. These demographic findings as presented further in Table 1, revealed that the study was based on interviews conducted with eight caregivers of adolescents who are living with HIV, and they were aged between 41 and 60. Seven of these caregivers were females, with only one male. On the employment front, only two participants were employed, and the rest were unemployed. Their educational profiles revealed that four participants attended school up to a secondary level, while the remaining four ended at a primary school level. None of the participants proceeded up to a tertiary level of education. On the question of the relationship that the caregivers had with the adolescent in their care, all of them were family members. Four were taking care of their biological children, two were grandmothers, and two were aunts. Six of the caregivers noticed the HIV-positive statuses of adolescents in their care when they began to fall sick, one discovered the status after the death of their mother, and the last one when the ALHIV was diagnosed with TB.

### 6.2. The Substantive Findings of the Study

Substantively, the findings of this study pointed to a number of experiences that were categorised into seven themes relating to regular monitoring of ALHIV who are always sick, the general behavioural issues of ALHIV, academic challenges faced by ALHIV, financial burden, lack of proper nutrition, and other issues, as well as the stigma and discrimination associated with caring for ALHIV. Table 2 below outlines these seven themes, which are further introduced and explained later.

#### 6.2.1. Theme 1: Regular Monitoring of ALHIV Who Are Often Sick

Based on the responses provided by the participants, it emerged that they encounter difficulties with monitoring the frequently sick ALHIV who are in their care. A total of five caregivers contributed to this theme, and C-VS was one of the participants who alluded to the challenges regarding the monitoring of her child who was always sick. This is what C-VS told researchers:

“*What I can say it is difficult to look after a child who is HIV because sometimes you want to give him his medication and he is still playing and now he is a teenager it is more difficult because sometimes he doesn’t want to take his medication, I will say it is difficult to look after such a child.*”

C-MO was another participant who indicated that regular monitoring through clinic visits was a challenge that interrupted her day-to-day functioning:

“*No really those are the challenges I am experiencing, just frequenting clinics, which is time consuming that is the reason I had to stop working so that I can be able to attend her clinic visits.*”

For C-TB, her ALHIV was sick from birth, and she was not aware that she was infected with HIV. And so having to monitor her condition came as a surprise:

“*He got sick, eish (is a South African phrase, used to express a wide range of emotions such as surprise, dismay, pain, admiration. It is similar to “wow,” “oh my goodness,” or “geez,”) when he was born, I didn’t know that I have HIV, when he was born, he couldn’t walk, so I took him to traditional healers thinking that he is bewitched, but it never helped, and my mom suggested that we take him to the clinic. Yeah, after taking him to the clinic that is how we found out that he is sick and they started giving him medication…*”

In the case of C-SF, the ALHIV was vomiting and required regular monitoring. This was frustrating to her, as indicated in the following extract:

“*Yeah, the problem is now she is vomiting, she has been vomiting for the past 3 years, all of a sudden, she started vomiting we don’t know what is causing it. She started in 2020, and she became weak because she vomits until her stomach is empty. We went to the clinic and at the clinic they gave us a note so she could go to the hospital… The vomiting and pain were affecting her school, but I encouraged her to continue focusing on taking the medication.*”

The burdensome roles of caregivers, as imposed by the diagnoses of the ALHIV, include regular ART clinic visits due to regular sickness and psychological care, which, as participants expressed, is time-consuming and makes it difficult to juggle daily activities. Some participants found it difficult to juggle the needs of their ALHIV and their employment, which in turn compromised their employment duties. Having insufficient time to spend on employment and income-generating activities often resulted in some caregivers being fired.

#### 6.2.2. Theme 2: The General Behavioural Issues of ALHIV

In addition to the demands associated with continuous monitoring of their ALHIV, six participants indicated that they were confronted by behavioural difficulties displayed by these adolescents.

One of the participants who voiced behavioural issues among ALHIV in this regard was C-TB, who stated the following:

“*You know the stage where they start getting smart, yes adolescent stage, eish I have to always reprimand her. When she has to come back home from school she comes back late, and when I ask, she will say she was with her friends. I keep on reprimanding, but she will change for few days and go back to her old ways.*”

Our interview with C-MJ also revealed sentiments involving ALHIV’s behavioural issues. This is how she articulated it:

“*Eish they* [ALHIV] *are difficult, they don’t understand, they think they know it all and on top of it, she has HIV so she can’t just date anyone she needs to understand that she can infect others with HIV. But she is blaming me.*”

In an interview with C-ID, she also alluded to problematic behaviours of ALHIV. This is what she had to say:

“*You know especially when they start being adolescents, they are troublesome because they start dating and they don’t know that they are HIV positive.*”

C-SF alluded to how the ALHIV in her care returned pregnant after visiting her father:

“*She is now 16 years old, you know in December she went to visit his father in Phalaborwa, and she came back pregnant and I worry that she will give birth to a child who has HIV.*”

C-VS indicated that she always had to remind her ALHIV to even take a bath. This is how she shared her predicament:

“*Yes, it is difficult you experience a lot of challenges like even taking a bath, if you don’t remind him, he will stay for 2 days without bathing, and he sometimes stay until late and when you ask him he will say he was with his friends. But I worry if he now has a girlfriend and he might infect the other child without thinking about it or make her pregnant.*”

C-DA also reflected problematic behaviours displayed by her ALHIV:

“*Mistakes like going to be with a friend and come home late after sunset. The sun shouldn’t set on a female child not being home…*”

Behavioural concerns such as risky sexual behaviours, lack of proper hygiene, and spending more time with friends result in being late and not fulfilling some duties. Primary caregivers are worried and stressed about the sexually risky behaviour of their ALHIV and their general misconduct. Apart from the negative impact of these behavioural issues on their own well-being, they also cause psychological stress and added burdens to the primary caregivers. The multiple risky behaviours of ALHIV negatively impact their well-being, particularly their health, and ultimately cause psychological stress and burden to their primary caregivers.

#### 6.2.3. Theme 3: Concerns About Failure of ALHIV to Comply with Their Treatment

Five participants in this study articulated their constant worries about the refusal of ALHIV to properly take their HIV treatment as prescribed. Caregivers were more concerned that poor uptake of HIV treatments by ALHIV might lead to poor health and have extreme repercussions.

C-TB was one of the participants who reported the difficulties adolescents had in adhering to treatment. Her concerns are reported below:

“*It is because her mother failed to monitor her, she was just saying to her take your pills and she will throw those pills away, ya that is how she stopped taking her treatment. Ya so when I usually visit, I decided to check (said the name of the granddaughter) clinic card and I saw the date on the card, got worried and I asked the mother when did (said the name of the granddaughter) go to the clinic and my daughter said why are you asking is there a problem. Then I told my daughter the card shows that she is not going to the clinic. My daughter said but she still has medication. When I ask my granddaughter, she will say I drink my pills but sometimes I forget.*”

C-DI, who was also concerned about the failure of ALHIV to adhere to treatment, had this to say:

“*My worry was acceptance of her HIV status, not wanting to take her medication, coming late from school and fail to take her medication on time…*”

Caregivers C-SF and C-MJ reported that their ALHIV experienced treatment fatigue and ended up stopping treatment:

“*It was hard because we started the medication when we got here in Joburg, because at home my mother couldn’t afford to run around and go to the clinic with a sick child while I’m busy looking for work. So, I decided to come to Johannesburg with my child and I brought her here to the clinic, here at the clinic they did everything, and the child started taking the medication and treatment in 2014. When she becomes an adolescent, she didn’t want to take the medication, she will say these things are bad, they make me vomit and I am tired. I told her that she needs to be strong we will report this at the clinic.*”

Then, caregiver C-MJ added:

“*He was 16 years, but he didn’t understand, sometimes he will say that I am tired of taking these pills, so my mom is also HIV positive, and she is taking these pills, so she will tell him that you can see that I am also sick and taking these pills, so if you don’t take them you will get sick and die. Sometimes he will stop taking his medication and he will start developing a rush all over his body and the face, so we will explain to him that if he continues to stop taking his medication his face will get worse with this rush. You know sometimes he doesn’t want to take the medication saying that it chokes him and can’t pass through his throat, so we have to give him sweets to be able to take them.*”

ART adherence among ALHIV was poor due to barriers to adherence, such as forgetting, needing a break from taking medications, and not wanting to be reminded of HIV.

#### 6.2.4. Theme 4: Academic Challenges Faced by ALHIV

The fourth theme that emerged from analysis of the participants’ responses pointed to the worries that caregivers have regarding the academic challenges faced by their ALHIV. A total of six caregivers responded to this theme. Among the caregivers who alluded to this challenge was C-MJ, who had this to say:

“*I also assist her with schoolwork because sometimes she is slow compared to other kids and sometimes missed school because of her illness.*”

An interview with C-DA also pointed to some worries about academic challenges:

“*Sometimes he gets sick, my child is now 18 years, and he is still in grade 8, I think he is slow I don’t understand. I was referred to a social worker in town and they promised to remove him from the current school to a new school and also at school they say he is not coping well.*”

For C-ES, the ALHIV had coping challenges after the death of her mother, which affected her academic activities. This is what C-ES told us:

“*Yes, she wasn’t coping at all after her mother’s passing, sometimes she will just be at school and cry, not do her homework or even sometimes not complete her schoolwork, and at school they had to take her to a lower grade.*”

In the case of C-SF, her ALHIV’s academic issues began when she started to get sick. She narrated her challenge as follows:

“*When she was doing Grade 11, she failed because she started getting sick, you know the vomiting issue but because she liked going to school, she insisted even when she is sick. She lost focus on her schooling because of being sick…*”

For C-TB and C-MO, their ALHIV’s academic activities were a result of behavioural problems. This is what C-TB had to say:

“*Yes, at school, she once gave a problem, she would go to school and not get into the school yard for 3 weeks, she will sit at the park near school. So, I received a call from the school saying she hasn’t been to class for 3 weeks. I called her mother who went straight to school to get more information.*”

Sentiments similar to C-TB were also echoed by C-MO, who attested by saying:

“*While busy with my brother I received a call from school that my sister’s younger child is absconding at school, and they want to see me and when I confront him, he says at school they got the wrong person. So, I followed up the following day at school, the teacher informed me that he comes to school, but he never gets to class, and they have been sending call letters, so it means they never reached home. It was difficult because all the teachers say he never gets to class, never does his homework or schoolwork and give them an attitude.*”

Pertaining to the above comments, participants were more worried about their ALHIV falling behind academically due to behavioural problems at school (such as absconding, bunking classes, and having a disrespectful attitude) and illness-related disruption. Other participants were concerned about the ALHIV’s school performance, which was affected by the death of a parent.

#### 6.2.5. Theme 5: Financial Burden

In addition to the above issues, as highlighted by the participants, all eight participants were also concerned about the financial burden. Regarding the financial burden, C-DI reported the following:

“*Yes, I do, you know sometimes it is difficult financially, but you know sometimes we will not be having money for bread, but God’s grace someone will come and help. Especially when we get to the 15th of the month it is hard, but we are grateful that we are alive. You know we are many in total I have 7 children, 4 are for my sister and 3 are mine, so we live on foster care grant, but now I am worried that (she mentioned the name of ALHIV) after giving birth she will be removed from the foster grant, ya but we will cross the bridge when we get there.*”

C-VS indicated that financial challenges normally result in a failure to meet the child’s needs. He explained it as follows:

“*Ya it is difficult you might find out that he has lot of needs that you are unable to meet like he might need money…*”

Another concern regarding financial burden emerged from C-TB:

“*I am struggling my granddaughter needs money to buy lunch at school and the school is far, so she also needs transportation. Transport to school is R30 a day so if I don’t have, I have to make a plan, and she always says granny I get hungry at school, so I need to give her that money and it is bad I don’t have it.*”

In the case of C-ES, she struggled with purchasing food and uniforms due to financial issues:

“*I was referring to the fact that we don’t have money, food and that my granddaughter doesn’t have enough school uniform, you know it is hard without money to take care of a child who is sick, sometimes no money to visit the clinic when she is sick, and her mother passed away so sometimes she becomes emotional, and his father is Pretoria. I do not qualify for a pension as yet, so I recently registered, and I am not working so getting adequate food for her is a problem more especially since my son stays in Pretoria.*”

For C-DA, it sometimes gets so financially difficult that sometimes they would sleep without food:

“*Eish ya, like I said at home were too many so sometimes we sleep without eating, so I must go hassle and look for food, so that he can eat and have money to buy lunch at school, at school the is a food scheme program so they give them food for free, but he is unable to eat it because he vomits, so I must make sure that he has R10 to buy fat cakes and he will be fine.*”

C-SF reported that financial challenges prevent her from providing things like school uniforms and private medical care:

“*I will be happy if I can be able to provide for her and give her healthy food so that she can be able to take her medication and be strong. Lack of money is also a problem because I am struggling sometimes to get her school uniform or to take her to a private doctor and end up going to the clinic. You know girls like to look beautiful and I can’t afford to buy her expensive clothes, and I hate to always ask from family members, I should be able to provide for my children, I need money, and working will make a difference in my life and I will stop begging.*”

According to the participants, financial issues make it difficult for them to meet the needs of ALHIV, such as transport costs to visit clinics to honour follow-up dates, the provision of food and clothing, and pocket money for school.

#### 6.2.6. Theme 6: Lack of Proper Nutrition and Other Means

Proper nutrition was highlighted as essential for a healthy life for PLHIV. This emerged from the interviews conducted with eight caregivers. Among participants who contributed to this theme was C-ES, who reported issues pertaining to proper nutrition for her ALHIV, stating that her ALHIV needs nutritious food.

This is how her narrative was captured:

“*Sometimes when she eats some of the food disturbs her stomach, so I always need to ensure that she eats healthy food and that won’t upset her stomach. Her tummy will run, she will have diarrhoea so I make sure that I don’t give her milk and oily food, but you know how children are she will sometimes eat chips and all the wrong food.*”

Another caregiver who alluded to nutrition-related challenges was C-VS:

“*Yes, it is difficult you might find out that he has lot of needs that you are unable to meet like he might need money, food, ya healthy food or clothes and you don’t have money, so he becomes angry at you.*”

The contribution made by C-SF on this theme was as follows:

“*I will be happy if I can be able to provide for her and give her healthy food so that she can be able to take her medication and be strong.*”

Issues pertaining to nutritious food also emerged during our interview with C-TB, who had this to say:

“*I mean it is difficult in terms of getting food, particularly from the middle of the month, eish my daughter buy food for R2000, but you know children misuse the food, they misuse the food shame. Ya it is difficult and frustrating, so sometimes I go look for money from illegal money lenders, ya it is difficult.*”

For C-DA, they are six in their household and nutritious food was a challenge:

“*Yes, food is a problem because we are 6 at home, but my son is getting a grant, a foster care grant. So, it is not enough but my mom and I try to add but the food won’t be enough to finish the month. My brother is wasteful when it comes to food, and he end up controlling us and telling us he is the owner because he is boy.*”

This part of the findings highlighted the difficulties in consistently providing healthy food due to limited resources, which hinders the ability to meet the nutritional needs of ALHIV, with the potential to result in poor adherence to HIV treatment by ALHIV. Expression of frustrations by the primary caregivers who were unable to provide for their ALHIV often led to tensions in their relationship.

#### 6.2.7. Theme 7: Stigma and Discrimination Associated with Caring for ALHIV

Another theme that emerged from the analysis was that caring for ALHIV attracts stigma and discrimination. Four caregivers expressed fear of being stigmatised, which they often experienced during the disclosures of HIV status by their ALHIV, as well as when they visited the healthcare facilities for treatment. C-DI was one of the caregivers who, due to HIV-related stigma, was unable to freely visit the clinic. This is how she narrated her story:

“*Yes, and sometimes I would feel ashamed and start asking myself what people were going to say about my child, but I realised that this thing is normal, anyone can have it so I had to accept.*”

Another caregiver who was concerned about stigma and discrimination was C-MO, who had this to say:

“*I meant fear that she will disclose her status to other children, when she was still young she use to know that she has germs that are making her sick and she is taking medication and she shouldn’t tell anyone, but later I was assisted by Friends for life to tell her that she is HIV positive which was difficult because she was angry with me and even stopped taking her medication. This was very difficult and stressful to me.*”

The participants were worried about what others would say regarding their adolescents who were living with HIV and expressed fear of disclosure to peers as a major stigma concern for the adolescents. The above narratives affirm the continued existence of HIV-related stigma, even among those who care for the victims.

## 7. Discussion

The overall aim of this study was to explore the lived experiences and challenges of the primary caregivers caring for ALHIV in Gauteng Province, South Africa. The demographic data of our study seem to be consistent with findings of other studies, which generally depict caregivers as women in their majority [58,59]. Since it is commonly women who seek assistance with childcare-related issues from government and NPOs, the domination of female caregivers in our study did not take researchers by surprise. In most African societies such as South Africa, caregiving is deemed to be the responsibility of women, though some men do participate under certain circumstances such as when willing to do so or when women are unable to do so due to reasons such as illness. It is also clear from our study that, caregivers navigate a complicated caring journey which is characterised by challenges such as the need for continuous monitoring of ALHIV who are always sick. This monitoring is time-consuming and negatively affects their employment and other income-generating activities. The complexity of monitoring ALHIV was also raised in other studies [11,60]. In the midst of these complex caregiving duties, caregivers sacrifice their other responsibilities of providing for the entire family in favour of the adolescents [11,60]. The microsystem, which is realised in the family or within the household, is characterised by challenges that place demands on both ALHIV and their family members, including primary caregivers. Considering this part of the findings from the microsystem of the ecological systems theory, it is essential to remember that an ALHIV is at the centre of attention and that his or her needs play a crucial role in shifting the normal roles of other family members (i.e., limiting the usual times that a caregiver would spend on generating income for the family in order to provide care).

The mesosystem considers the interactions between systems such as primary caregivers, employers, school or healthcare systems. The conditions of ALHIV often require renegotiations with external systems such as the schools to allow them time-off so that they can access their treatment while caregivers also have to negotiate the same with employers in order to care for their adolescents when they fall sick. This reflects the influence of the interactions and interpersonal relations on human development across the micro-, meso-, exo-, and macrosystems [36]. Several scholars found results, in alignment with our findings, revealing that ALHIV are likely to have social problems and/or multiple risky health behaviours including poor adherence to treatment, with the potential to negatively affect their well-being and cause significant risks and burdens to their families [61,62,63]. The critical role that nurses can play in supporting primary caregivers and ALHIV is to address the challenges pertaining to treatment adherence and status disclosure.

Since nurses are a first point of contact within healthcare facilities, they are strategically positioned to provide continuous psychosocial support, health education, and adherence counselling to both ALHIV and their caregivers [64,65]. Nurses can also play an essential role in supporting ART adherence among ALHIV which, as demonstrated by this study, was poor due to barriers such as forgetfulness, needing a break from taking medications, and not wanting to be reminded of HIV. In such instances, nurses could coordinate educational programmes to empower both adolescents and their caregivers with regard to the importance of treatment adherence. Such educational interventions could be executed through the use of peers as ambassadors of healthcare among the adolescents.

The exosystem of the ecological systems theory consider structures such as neighbourhoods and friends to be instrumental in either negatively or positively influencing ALHIV and their behaviour [27,28,30]. Behavioural issues of ALHIV will undoubtedly affect some areas of their lives; hence, the findings of our study also pointed to poor adherence to HIV treatment. Our findings seem to support some existing studies [11] based on the experiences of caring for ALHIV indicating that they sometimes refuse to take their HIV medication and fail to attend clinic visits, leading to poor adherence to antiretroviral therapy (ART). Although South Africa has had a successful roll-out of HIV treatment and several interventions to improve adherence, ALHIV are reported to have a poor adherence record compared to adults and children [58].

Poor academic performance among ALHIV is another challenge reported by participants, and it seems not to be a new phenomenon. Several researchers have also demonstrated that ill-health and cognitive challenges are associated with living with HIV [55,56,59,60]. Reading this part of the findings in the context of an ecological systems’ perspective, a clear demonstration of the impact of a mesosystem, particularly the relationship between a person’s specific microsystems (in this instance, the school), and how they positively or negatively influence the individual by working together or against each other is highlighted [66,67,68]. In drawing from the findings of this study and the literature, it is evident that ALHIV are negatively affected by their illness, while at the same time being exposed to mental health-related and other issues. Moreover, regular visits to health facilities, which consequently lead to absenteeism at school and eventually poor academic performance, are also common.

Financial burdens and a lack of food or infrequent meals seem to be common difficulties confronting caregivers of ALHIV. Such were also reported by several researchers [64,65], who indicated that financial difficulties faced by primary caregivers extend to transport costs for visiting healthcare centres to honour scheduled appointments. All these challenges confront caregivers who often lack the necessary financial resources to at least respond to some of the ALHIV’s needs, such as providing adequate food in order to support treatment uptake. Financial difficulties are also linked to employment, mental health, and the general state of poverty among the caregivers, who are burdened by employment and financial concerns that negatively affect their mental and physical health [69]. Poverty results in a lack of proper meals, clothing, and school fees for ALHIV [7,69], which in turn leads to poor attendance of their clinic appointments and missing appointments due to a lack of transportation money. The dire financial situation of caregivers reflected broader socioeconomic challenges. Some of the caregivers were from impoverished and underprivileged communities that are characterised by unemployment and a lack of financial resources to meet the needs of ALHIV and their families. Notwithstanding the above, South Africa has systems such as the Social Security Agency (SASSA) and a few NGOs in place to support people with difficulties such as those reported by the participants. However, the assistance provided to these caregivers, adolescents and other beneficiaries of these services is not always adequate to address their needs. For some caregivers, financial support was sometimes provided by family members, which made it easier to meet some of their needs [65,70].

Considering this part of the findings from the ecological theory, factors such as the socioeconomic status (microsystem) of primary caregivers and a lack of resources, such as transport money to go to school or the clinic, or even food to eat in the family, may negatively impact the health condition of ALHIV and their primary caregivers [71,72,73,74,75]. Writing on Maslow’s hierarchy of needs, Stefan et al. argue that an individual’s motivation rests on a hierarchy of needs, one of which is physiological and security needs, which are shelter, food, sleep, and other basic needs for human survival [76]. A Kenyan study of food security, parenting practices, and caregiver–adolescent relationships revealed that income-generating interventions may assist in the provision of food and improve food security within the family and therefore contribute positively to parenting practices and the psychosocial well-being and behaviour of adolescents [77]. The basic needs of primary caregivers are therefore paramount, and failure to meet them might result in stress and other mental health issues, which may, in turn, negatively affect the health conditions of ALHIV.

As demonstrated by our study, more than four decades into the HIV pandemic caregivers still face stigma and discrimination. Previous researchers also found that caregivers are subjected to stigma by virtue of being associated with caring for PLHIV [78,79,80]. The danger of stigma is that it can prevent disclosures of HIV-positive diagnoses and discourage people from seeking information due to fear of being perceived as living with HIV [76]. To primary caregivers of ALHIV, this may have far-reaching consequences, including the stress associated with the fear that the HIV diagnosis of an ALHIV will be known and that they might be subjected to stigma and discrimination. What was disheartening, which is also found in some parts of the existing literature, is reports by participants that stigma and discrimination associated with caring for ALHIV were also perpetuated by some members of their own families and communities, which negatively impacted ALHIV’s access to treatment [26,81,82]. However, it is also encouraging that some primary caregivers experience less stigma and discrimination and more support particularly from their family members [11,70].

Considering the challenge of stigma as experienced by the participants from an ecological systems theory, it is crucial to remember that stigma should not only be understood narrowly as an impact on caregivers and ALHIV. It is driven by factors embedded in other societal systems, such as their families and their communities, and it is sometimes even indirectly promoted through economic factors, policies, societal cultures, and practices. Some of these systems carry negative factors, such as HIV-related stigma and discrimination influenced by a variety of social, political, economic, and cultural factors [36,82,83,84]. Therefore, in order to effectively respond to stigma, it is essential to address these factors from all other systems, including by empowering the caregivers on how best to cope with stigma and by educating family members and communities about the dangers of stigma. Therefore, the findings of this study should be understood in the broader context of the socioeconomic situation of South African society. HIV is clearly more than a health issue affecting ALHIV. The relationships that ALHIV have with their friends either shape their behaviour to be acceptable or unacceptable in the eyes of their caregivers. The caregivers’ activities, including formal or informal employment, may be cut short or suspended in their attempts to care for and support their ALHIV. The state of these adolescents’ health, on the other hand, affects their academic activities, with a high likelihood of caregivers being called into school every time they miss school or perform poorly in class activities. Compounding these issues is the stage in which these children find themselves, which is the adolescent stage, described by some authors as characterised by intense physical, cognitive, mental, social, and behavioural factors that often result in mental health-related issues [85]. These challenges have the potential to negatively hamper the caring roles of caregivers, which may in turn affect access and compliance to treatment, care, and support by ALHIV [11,65]. Therefore, the challenges that caregivers and their ALHIV struggle with on a daily basis, as revealed by this study, indicate that governments and civil society need to focus on intensifying efforts to effectively deal with HIV and its related impact on ALHIV and their caregivers. Failure to intensify the fight against HIV and its related impact may lead to the broader societal targets of preventing HIV and supporting those living with it being a distant goal.

## 8. Conclusions

This study sought to explore the lived experiences and challenges of primary caregivers caring for ALHIV in Gauteng Province, South Africa. The interviews conducted with the caregivers of ALHIV, as guided by qualitative research, led to the discovery of a number of challenges encountered by these caregivers. Caregivers tend to battle with navigating their own activities and monitoring these youngsters, who are often sick and require care and support through healthcare facilities. The complexities encountered by these caregivers as they navigate through their caregiving trajectory include having to deal with behavioural issues and academic challenges displayed by these ALHIV. Further compounding these issues for caregivers is financial challenges, which often translate into a lack of, or inadequate, food within their households. Despite interventions for addressing stigma having been in existence for as long as four decades, stigma and discrimination remain common in communities, negatively affecting efforts towards caring for and supporting these ALHIV, as well as preventing further spread of the virus. For these caregivers, caring for an ALHIV is not only limited to their relationship, but it is a complex task cutting across their workplaces, the school environment, and their community.

Given the impact of caring for ALHIV, as demonstrated by this study, most of the primary caregivers were unemployed and therefore experienced financial difficulties. It is therefore recommended that they should be linked to financial support programmes and agencies such as the South African Social Security Agency for state-provided social grants. In terms of the need to continuously monitor the health of ALHIV, caregivers who are employed may be at risk of losing their jobs. It is therefore essential for professionals, such as social workers, to extend their support to the workplaces of these caregivers to lobby employers into the value of supporting societal challenges regarding HIV. Community-based multisectoral forums comprising social workers, nurses, educators, businesses, and other key stakeholders should be initiated as a structure to support HIV programmes in communities. Such will contribute towards informing the community on HIV-related issues and therefore mitigate stigma and discrimination while ensuring that those living with HIV receive necessary support. Some recommendations for future studies are proposed, including the inclusion of a larger sample and primary caregivers who are non-family members, as well as covering other provinces in order to develop comprehensive insight into the experiences of caregivers. It is also recommended that further studies be conducted to deliberately include caregivers from other racial groups and genders, as well as those from economically disadvantaged communities.

## 9. Limitations

Despite having contributed to the state of knowledge and literature around the issue of caregivers and ALHIV, this study had its limitations, and they are as follows:

- The sample size was relatively small due to the sensitivity of the topic; interpretation of the findings should therefore be done in consideration of this small sample.
- All participants were based in the province of Gauteng (City of Johannesburg, Tshwane, Ekurhuleni, and Sedibeng), which is predominantly urban in outlook. Data from other provinces could possibly provide a different picture.
- All primary caregivers were family members (such as parents, grandmothers, and aunts), which excluded non-family members as primary caregivers. The possibility that caregivers who are non-family members or relatives may provide a different picture cannot be ruled out.
- Most of the participants were black females who were receiving services from NPOs that were rendering services to caregivers of children and adolescents from poor communities. This could be a limitation, since the experiences of male caregivers, women from other races and those from economically viable communities are left undocumented.

## Figures and Tables

**Table 1:** Demographic profiles of the participants.

Participant	Age	Gender	Ethnicity	Employment	* Education	Relationship	Disclosure About ALHIV Status
C-MJ	46	F	Tsonga	Unemployed	Grade 11	Aunt	When child fell sick
C-DI	41	F	Zulu	Unemployed	Grade 7	Mother	When child fell sick
C-ES	60	F	Swati	Unemployed	Grade 5	Grandmother	After the mother’s death
C-VS	56	M	Tsonga	Unemployed	Grade 5	Father	After TB diagnosis
C-SF	53	F	Xhosa	Unemployed	Grade 5	Mother	When the child fell sick
C-TB	57	F	Zulu	Unemployed	Grade 5	Grandmother	When the child fell sick
C-DA	53	F	Xhosa	Unemployed	Grade 5	Mother	When the child fell sick
C-MO	46	F	Pedi	Unemployed	Grade 5	Aunt	When the child fell sick

* The education profile displayed in this table is that of the caregivers.

**Table 2:** Themes.

Theme	Description
Theme One	Regular monitoring of ALHIV who are always sick
Theme Two	The general behavioural issues of ALHIV
Theme Three	Concerns about failure by ALHIV to comply with their treatment
Theme Four	Academic challenges faced by ALHIV
Theme Five	Financial burden
Theme Six	Lack of proper nutrition and other means
Theme Seven	Stigma and discrimination

## Data Availability

The data presented in this study are available on request from the corresponding author due to data restrictions imposed by South Africa’s Protection of Personal Information Act No. 4 of 2013 (POPIA).

## Public Involvement Statement

No public involvement in any aspect of this research.

## Guidelines and Standards Statement

This manuscript was drafted in accordance with the ENTREQ.

## Abbreviations

The following abbreviations are used in this manuscript:

ALHIV	Adolescent living with HIV
ARV	Antiretroviral
UNAIDS	Joint United Nations Programme on AIDS
UNICEF	United Nations Children’s Fund
HSRC	Human Sciences Research Council
SANAC	South African National AIDS Council
HIV	Human immunodeficiency virus
NPO	Non-profit organisation
OVCAY	Orphans and vulnerable children, adolescents and youth
COVID-19	Coronavirus disease
PLHIV	People living with HIV
